# Ultrasound‐Guided Percutaneous Sclerotherapy With Bleomycin for Management of Infantile Subcutaneous Hemangioma: A Case Report

**DOI:** 10.1002/ccr3.70143

**Published:** 2025-02-11

**Authors:** Iman Kiani, Mitra Khalili, Fahimeh Abdollahimajd, Peyman Eshghi, Arash Khameneh Bagheri

**Affiliations:** ^1^ Students' Scientific Research Center Tehran University of Medical Sciences Tehran Iran; ^2^ Department of Radiology Shahid Beheshti University of Medical Sciences Tehran Iran; ^3^ Skin Research Center Shahid Beheshti University of Medical Sciences Tehran Iran; ^4^ Pediatric Congenital Hematologic Disorders, Research Institute for Children Health Shahid Beheshti University of Medical Sciences Tehran Iran

**Keywords:** bleomycin, hemangioma, sclerotherapy, ultrasound‐guided intervention

## Abstract

Percutaneous sclerotherapy with bleomycin is an effective, minimally invasive treatment for aggressive pediatric hemangiomas, especially when traditional therapies fail. This approach can improve clinical outcomes, including thrombocytopenia resolution and tumor size reduction, with minimal systemic side effects.

## 1 Introduction

Hemangiomas are the most common benign tumors in infants, characterized by their rapid growth phase followed by a slow involution period that typically resolves by age 10 [1]. Despite their benign nature, approximately 10% of hemangiomas require intervention due to complications such as ulceration, bleeding, or life‐threatening growth patterns [2]. Hemangiomas are classified broadly into infantile hemangiomas and congenital hemangiomas. Although both represent vascular anomalies, they differ significantly in terms of onset, clinical behavior, and histological features. Infantile hemangiomas typically present within the first few weeks of life, characterized by a rapid proliferative phase during the first year followed by a gradual involution phase over the subsequent years, while congenital hemangiomas are fully developed at birth and do not follow the typical growth and involution phases of infantile hemangioma [3].

Traditionally, management options for complicated hemangiomas include corticosteroids, beta‐blockers like propranolol, and, in more severe cases, chemotherapeutic agents such as vincristine [4]. However, these systemic treatments can be associated with significant side effects and variable efficacy [5]. In recent years, image‐guided angioembolization has emerged as a promising alternative, which offers targeted therapy with a high degree of precision and minimal systemic involvement [6]. This technique involves the selective occlusion of abnormal blood vessels supplying the hemangioma, resulting in reduced tumor size and amelioration of symptoms. However, the application of angioembolization in pediatric patients is challenging due to the small size and fragility of their vascular structures and also for posing the risk of hemodynamic instability [7]. Consequently, percutaneous sclerotherapy, specifically with bleomycin, emerges as a preferable alternative [8]. While this method has mostly been applied in hepatic hemangioma [9], here we review a successful application of ultrasonography‐guided percutaneous sclerotherapy in treating a case of hemangioma. Of note is that written informed consent was obtained from the parents of the child for publishing the report.

## 2 Case History/Examination

**FIGURE 1:**
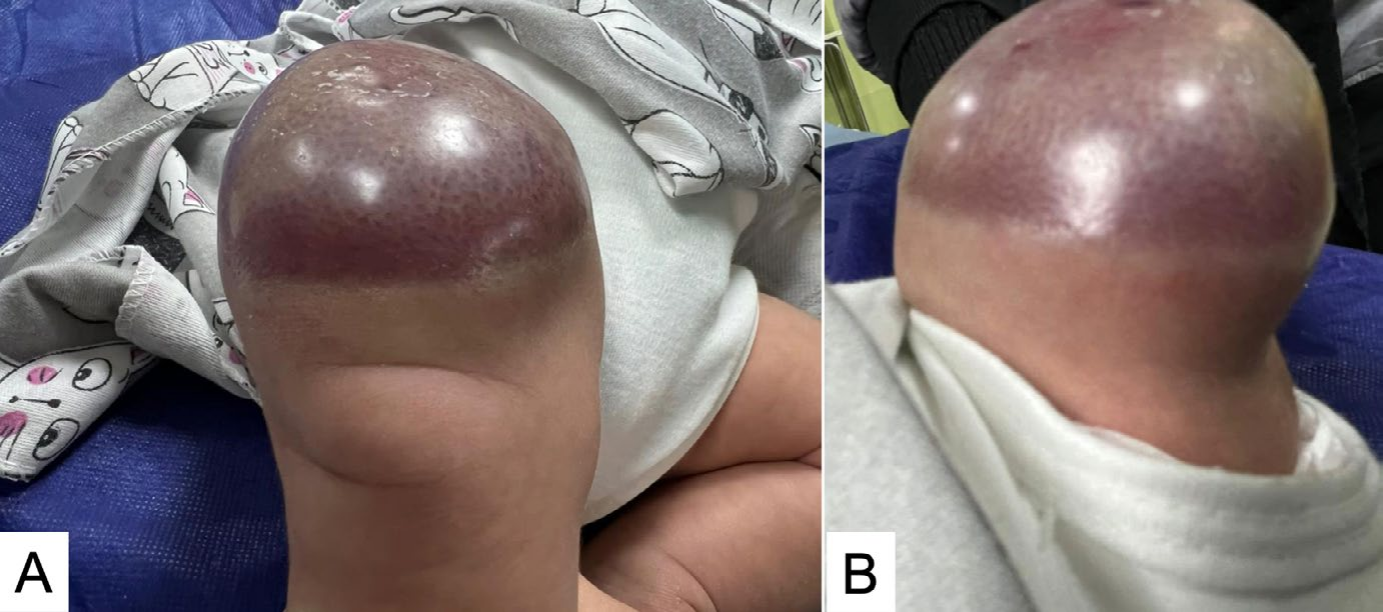
Clinical presentation of the patient with a subcutaneous hemangioma located on the right thigh and knee.

**FIGURE 2:**
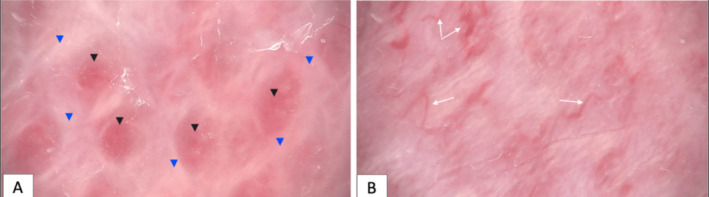
Dermoscopic images of the lesion. Black triangles: Red‐white homogeneous area; blue triangles: “white rail” lines; white arrows: Linear vessels.

This case report details the hematological and radiological findings in a 44‐day female patient diagnosed with a subcutaneous hemangioma located in the right thigh and knee (Figure 1). Regarding the laboratory results, the initial hematological evaluation revealed a decreased RBC at 2.33 × 10^6/μL and hemoglobin concentration at 7.4 g/dL, both indicative of anemia. The patient also presented with severe thrombocytopenia, with a platelet count critically low at 24 × 10^3/μL and slightly abnormal red cell distribution width (RDW‐CV) at 20%. Coagulation profiles showed a prothrombin time (PT) of 13.0 s, within normal limits, and a partial thromboplastin time (PTT) slightly elevated at 45 s, suggesting mild coagulation dysfunction (Table 1). Figure 2 presents dermatologic findings of the lesion, which collectively showed dense capillary structures with fibrotic changes.

**TABLE 1:** Laboratory results of the patient.

Parameter	Result	Unit	Normal range
WBC	8.5	×10^3/μL	4.0–10.0
RBC	2.33	×10^6/μL	M: 4.5–6.3, F: 4.0–5.5
Hemoglobin	7.4	g/dL	M: 14–18, F: 12–16
Hematocrit	22.1	%	M: 40–54, F: 36–48
MCV	94.8	fL	80.0–100.0
MCH.	31.8	pg	27.0–34.0
MCHC.	33.5	g/dL	31.0–37.0
Platelets	24	×10^3/uL	150–450
RDW‐CV	20.0	%	11.5–14.5
PT	13.0	s	12–16
INR	1.0		
PTT	45.0	s	28–45
D‐dimer	8589.0	ng/mL	< 500

Abbreviations: MCH, mean corpuscular hemoglobin; MCHC, mean corpuscular hemoglobin concentration; MCV, mean corpuscular volume; PT, prothrombin time; PTT, partial thromboplastin time; RBC, red blood cells; RDW‐CV, red cell distribution width; WBC, white blood cells.

**FIGURE 3:**
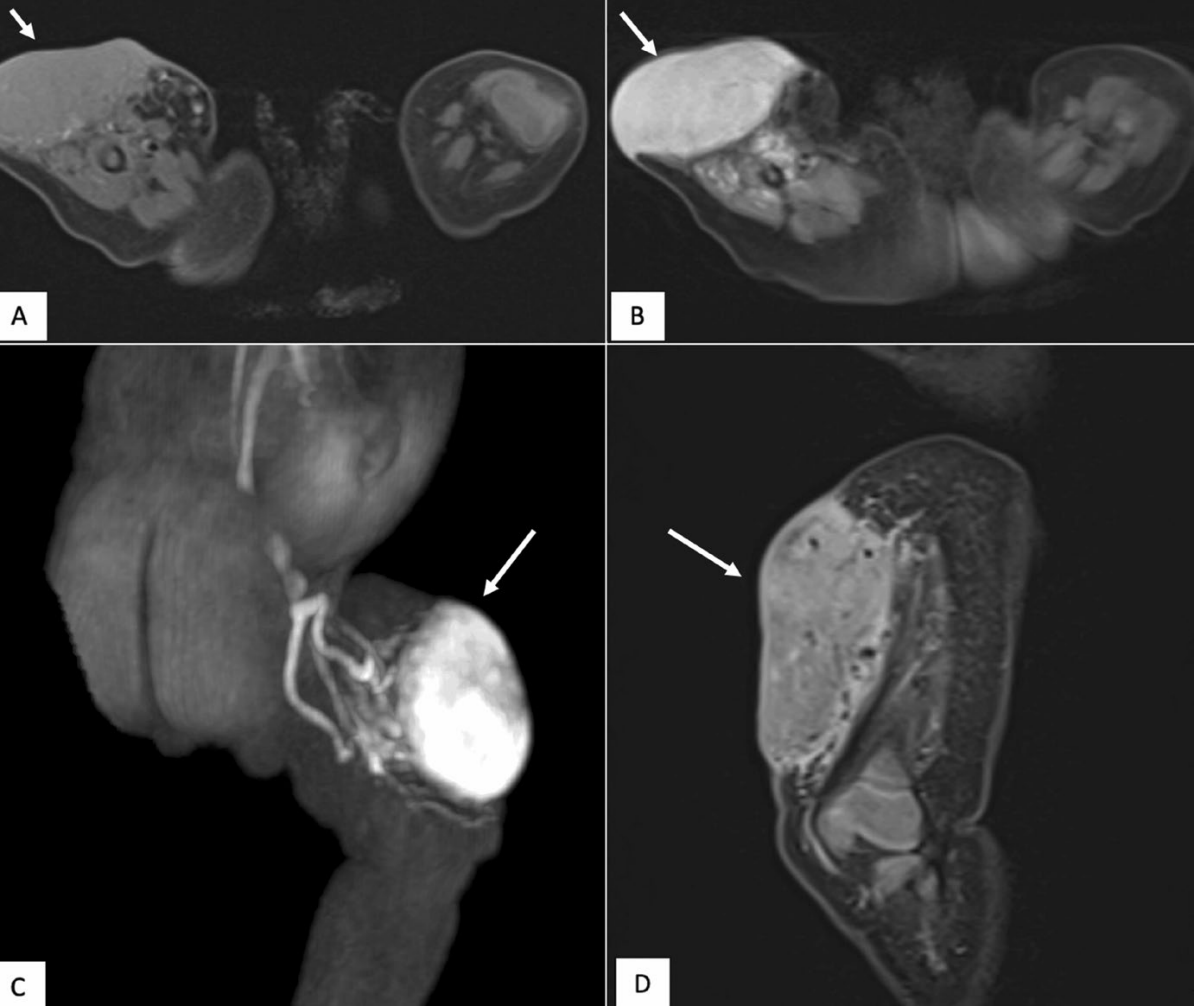
MRI Imaging of Subcutaneous Hemangioma in the Right Thigh and Knee. The white arrow indicates the lesion observed. A: Axial T1‐weighted MRI without contrast, showing a hyposignal lesion in the right thigh. B: Axial T2‐weighted fat‐saturated MRI with contrast, demonstrating enhancement of the lesion. C: Magnetic Resonance Angiography (MRA) in the sagittal plane, which shows feeding arteries. Signal void areas indicate high flow in the lesion. D: Sagittal T2‐weighted MRI with contrast, which shows hyperintensity within the lesion and its infiltration into the underlying quadriceps muscles.

MRI of the right thigh and knee was taken as well, which identified a subcutaneous tumor measuring 56 × 31 × 52 mm. The mass demonstrated T1 hypointensity and T2 hyperintensity, with multiple peripheral signal void structures consistent with vascular channels typical of hemangioma. The mass also exhibited infiltration into the underlying quadriceps muscles (Figure 3). These findings resembled kaposiform hemangioendothelioma (KHE), which is recognized as an aggressive form of vascular tumor typically occurring during infancy and is associated with significant morbidity due to its invasive nature [10].

## 3 Methods (Differential Diagnosis, Investigations and Treatment)

Regarding the management, initially, the patient was treated with sirolimus, a standard chemotherapeutic agent used for managing complex vascular anomalies by inhibiting mTOR pathways that promote vascular growth and proliferation [11]. Despite ongoing treatment, there was no significant change in the size of the hemangioma, and the patient's condition was complicated by persistent, progressive thrombocytopenia. Therefore, after confirming the absence of venous drainage through ultrasound‐guided compression of the drainage vein, percutaneous sclerotherapy with bleomycin was selected as the treatment modality for the infant's subcutaneous hemangioma. Two milligrams of bleomycin, diluted with 4 mg of triamcinolone, was administered directly into the hemangioma through four puncture sites under the guidance of ultrasound.

## 4 Conclusion and Results (Outcome and Follow‐Up)

**FIGURE 4:**
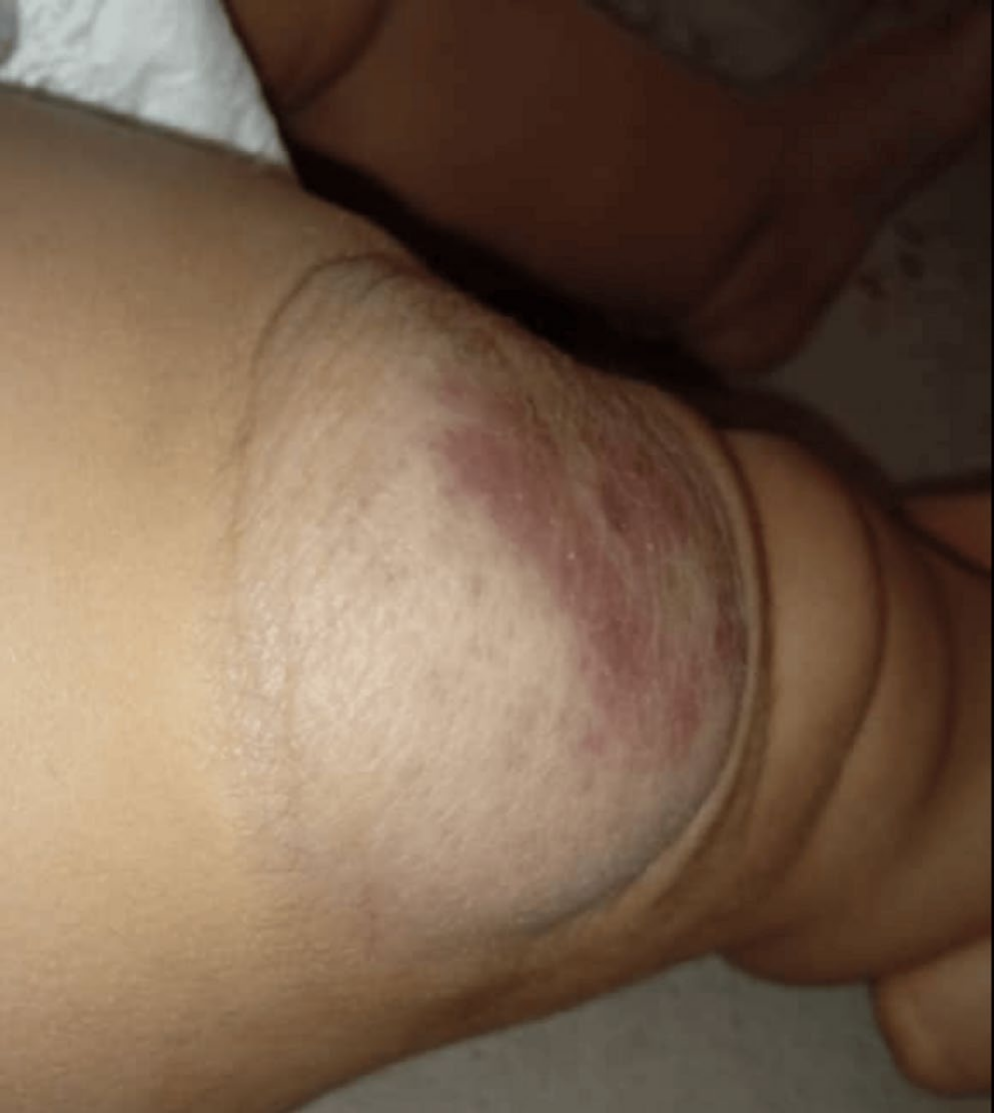
Post‐treatment status of the hemangioma following ultrasound‐guided percutaneous sclerotherapy with bleomycin. The image shows a significant reduction in the size of the lesion, with improved skin texture and color.

After the procedure, the patient was monitored closely for any immediate complications, such as swelling, pain, or signs of ischemia in the tissue surrounding the hemangioma. Follow‐up visits were scheduled to assess the long‐term efficacy of the sclerotherapy and to monitor for potential recurrence or reduction of the hemangioma. One week after the treatment, the platelet count increased to 150,000. Follow‐up images of the lesion, taken 5 months after the single‐session procedure, demonstrate the treatment's effectiveness (Figure 4).

## 5 Discussion

In this case, we reported a successful application of percutaneous sclerotherapy in a 44‐day infant. Percutaneous sclerotherapy has emerged as a valuable technique in the management of complex vascular anomalies, particularly in pediatric populations [12, 13, 14, 15]. This approach involves the direct injection of sclerosing agents into abnormal blood vessels, effectively reducing the risk and severity of complications associated with hemangiomas, such as excessive bleeding [16]. Traditional treatment modalities for hemangiomas, such as corticosteroids, beta‐blockers, and, in severe cases, chemotherapy agents such as vincristine, while effective, carry the risk of systemic side effects [16]. In contrast, sclerotherapy directly targets the hemangioma's blood supply, which results in rapid symptom relief and a decrease in hemangioma size, which can be critical in preventing life‐threatening complications such as Kasabach‐Merritt syndrome [17].

Moreover, the choice of bleomycin over other sclerosing agents is supported by recent studies suggesting that this agent achieves similar volume reductions in vascular malformations while maintaining a lower complication rate compared to other agents, such as ethanol [18]. Ethanol, while effective, is associated with higher rates of adverse effects, including severe pain, skin ulceration, and systemic toxicity. In contrast, bleomycin typically results in minimal and transient adverse reactions, such as localized swelling and fever, with a complication rate reported between 0% and 20% [19, 20].

The broader implications of percutaneous sclerotherapy are also noteworthy. Previous studies on the use of percutaneous sclerotherapy have mainly focused on its role in hepatic hemangiomas and have found promising results [9, 16]. Additionally, application of percutaneous sclerotherapy in the treatment of other infantile disorders, such as infantile cysts [21, 22], venous malformations [14, 23], and aneurysms [24, 25], have had favorable outcomes as well. Despite these advantages, the requirement for ongoing monitoring is emphasized, as some hemangiomas may require repeated treatments to maintain results. Moreover, the success of sclerotherapy can depend heavily on the hemangioma's response to initial treatment, which is influenced by its anatomical and physiological characteristics.

In addition, it is important to acknowledge that while sclerotherapy is less invasive than surgical interventions, it is not free of risk. Complications can include allergic reactions to the sclerosing agent, localized infection, or tissue necrosis around the injection site [26]. Immediate post‐procedural care is necessary for identifying and managing these risks. Long‐term care following sclerotherapy involves regular monitoring to detect any recurrence or growth of the hemangioma.

Furthermore, the technique of percutaneous sclerotherapy demands considerable expertise and precision in administration [27]. Not all treatment centers may possess the necessary technological resources or personnel with the requisite expertise in ultrasonography‐guided interventions, which potentially restricts the widespread adoption of this technique. Nevertheless, the successful application in specialized settings shows that this approach could be expanded.

In conclusion, percutaneous sclerotherapy represents a significant advancement in the management of pediatric hemangiomas, providing a targeted, effective treatment option with the potential for immediate and impactful results. As with any medical intervention, the success of sclerotherapy relies on careful patient selection, procedural execution, and post‐treatment care. Continued research and clinical studies with rigorous methodology are needed to further refine this technique, reduce associated risks, and broaden its applicability.

## 6 Conclusion

In conclusion, this case shows the effective application of percutaneous sclerotherapy using bleomycin for the treatment of a complicated hemangioma in an infant. The approach demonstrated significant improvements in the patient's condition, including normalization of platelet counts and a remarkable reduction in the size of the hemangioma. This minimally invasive offers an alternative to more invasive procedures like angioembolization. Nevertheless, more studies are needed to assess the efficacy and outcomes of this procedure.

## Author Contributions

**Iman Kiani:** investigation, writing – original draft. **Mitra Khalili:** methodology, validation, writing – review and editing. **Fahimeh Abdollahimajd:** supervision, writing – review and editing. **Peyman Eshghi:** supervision. **Arash Khameneh Bagheri:** conceptualization, project administration, supervision, writing – review and editing.

## Consent

Written consent was obtained from the patient's legal guardians for publication of the case details and accompanying images.

## Conflicts of Interest

The authors declare no conflicts of interest.

## Data Availability

The authors have nothing to report.
